# Safety of antithrombotic therapy in hereditary hemorrhagic telangiectasia: results from a prospective cohort study

**DOI:** 10.1016/j.rpth.2026.106943

**Published:** 2026-09-03

**Authors:** Luigi Di Martino, Fabiana Agostini, Daniela Feliciani, Barbara Funaro, Annarita Barberio, Rosa Talerico, Antonio Gasbarrini, Roberto Pola, Eleonora Gaetani

**Affiliations:** 1 Hereditary Hemorrhagic Telangiectasia Center, Fondazione Policlinico Universitario Agostino Gemelli IRCCS, Rome, Italy; 2 Unità Operativa Complessa di Medicina Interna, Ospedale Cristo Re, Rome, Italy; 3 Unità Operativa Complessa of Internal Medicine and Gastroenterology, Fondazione Policlinico Universitario Agostino Gemelli IRCCS, Rome, Italy; 4 Percorso Trombosi, Department of Aging, Orthopedic, and Rheumatologic Sciences, Fondazione Policlinico Universitario Agostino Gemelli IRCCS, Rome, Italy; 5 Department of Translational Medicine and Surgery, Università Cattolica del Sacro Cuore, Rome, Italy

**Keywords:** antithrombotic therapy, bleeding risk, epistaxis, hereditary hemorrhagic telangiectasia, prospective cohort study, venous thromboembolism

## Abstract

**Background:**

Hereditary hemorrhagic telangiectasia (HHT) is a vascular disorder characterized by a predominant bleeding phenotype. However, patients are also at risk of thrombotic events and may require antithrombotic therapy (AT), for which safety data remain limited.

**Objectives:**

To prospectively evaluate the safety of AT in patients with HHT using standardized longitudinal assessment of bleeding outcomes.

**Methods:**

We conducted a prospective observational study at a tertiary HHT referral center. Patients with a definite diagnosis of HHT and a new indication for antiplatelet or anticoagulant therapy were enrolled. The primary outcome was the incidence of hemorrhagic events, classified according to the International Society on Thrombosis and Haemostasis criteria. Secondary outcomes included worsening of epistaxis assessed by the Epistaxis Severity Score, changes in hemoglobin levels, and transfusion requirements.

**Results:**

A total of 33 patients (mean age 65.8 ± 12.9 years; 69.7% male) were included, for a total of 41 AT courses. During a mean follow-up of 21.1 ± 19.2 months, 2 major bleeding events (4.9%) occurred, both during anticoagulant therapy. Three clinically relevant nonmajor bleeds and one minor bleed were recorded. Worsening of epistaxis occurred in 24.4% of treatment courses, but mean Epistaxis Severity Score values did not differ from baseline. Hemoglobin levels and transfusion requirements were not significantly affected.

**Conclusions:**

In this prospective cohort, AT was associated with an acceptable safety profile in selected patients with HHT managed in a multidisciplinary setting. These findings strengthen the currently limited prospective evidence supporting individualized antithrombotic management in HHT, although larger multicenter prospective studies remain warranted.

## Essentials

• People with hereditary hemorrhagic telangiectasia may need antithrombotic therapy (AT) despite an increased bleeding tendency.
• We prospectively followed 33 patients receiving 41 antithrombotic treatment courses.
• Overall bleeding burden remained stable during AT.
• AT was generally well tolerated in this prospective hereditary hemorrhagic telangiectasia cohort.

## Graphical abstract

**Figure:**
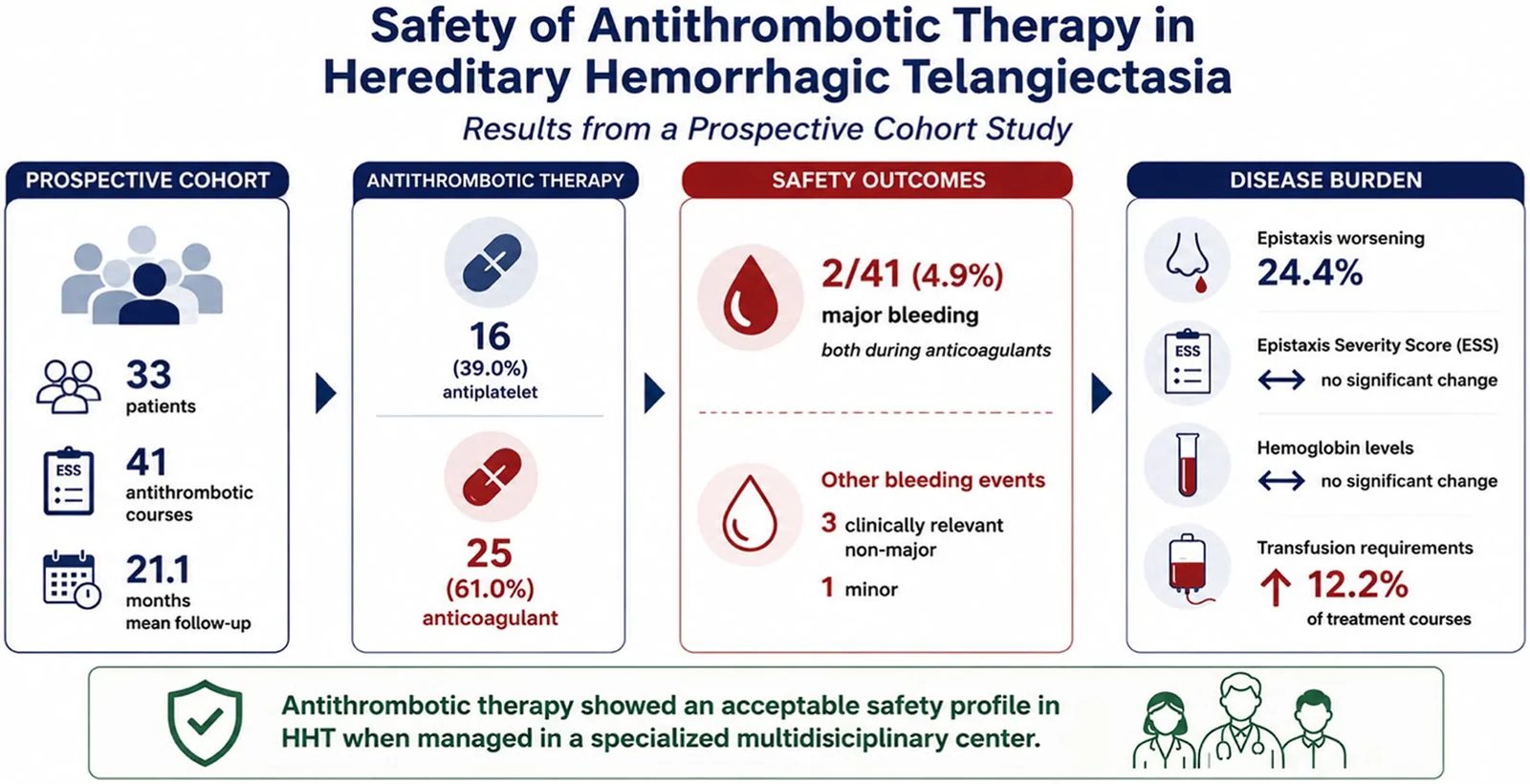

## 1 Introduction

Hereditary hemorrhagic telangiectasia (HHT) is an autosomal dominant vascular disorder characterized by abnormal angiogenesis leading to mucocutaneous telangiectases and visceral arteriovenous malformations (AVMs) [1,2]. Traditionally, HHT has been considered a rare disease with an estimated prevalence of ∼1 in 5000 to 8000 individuals [3]. However, recent genomic-based analyses have suggested that the true prevalence may be significantly higher than currently recognized, with a possibly considerable degree of underdiagnosis [4]. Clinically, the disease is marked by a predominantly hemorrhagic phenotype, with recurrent spontaneous epistaxis, chronic anemia, and bleeding from the gastrointestinal (GI) tract. AVMs in the central nervous system may be an additional source of critical bleeding [5,6].

Nonetheless, patients with HHT are not free from thrombotic conditions, including venous thromboembolism, atrial fibrillation, and arterial ischemic events [[7], [8], [9], [10]]. Importantly, recent prospective data have strengthened this concept, demonstrating a measurable incidence of thrombotic events in patients with HHT during longitudinal follow-up [11]. Consequently, it is not uncommon to have patients with HHT who require antithrombotic therapy (AT). The management of such therapy is particularly challenging in these patients due to inherent predisposition to bleeding. The Second International HHT Guidelines, published in 2020, recommend that anticoagulant or antiplatelet therapy should not be withheld solely on the basis of HHT when there is a clear clinical indication, while emphasizing individualized risk assessment and avoidance of combined or dual antithrombotic regimens whenever possible [12]. Despite these recommendations, the evidence supporting the safety of AT in HHT is limited and largely derived from retrospective studies. A European Reference Network on Rare Multisystemic Vascular Diseases (VASCERN) study reported worsening epistaxis in most patients with HHT treated with direct oral anticoagulants, with a high rate of treatment discontinuation [13]. Similarly, data from the Registro Informatizado de Enfermedad Tromboembólica (RIETE) registry showed that bleeding events during anticoagulation were not uncommon in patients with HHT treated for venous thromboembolism [14]. More recent retrospective studies have expanded the available evidence. A multicenter cohort study published in 2023 reported that approximately half of patients with HHT required dose reduction or discontinuation of AT due to bleeding complications, with an associated decline in hemoglobin levels and increased transfusion requirements [15]. Consistently, the French RETROPLACOTEL cohort identified early bleeding risk—particularly within the first 3 months of therapy—and highlighted prior bleeding history, age, and recent transfusions as key predictors of poor tolerance [16]. A contemporary scoping review confirmed that worsening bleeding is frequent and that treatment discontinuation occurs in a substantial proportion of patients [17].

Overall, current evidence remains limited by retrospective design, heterogeneity of patient populations, and lack of standardized outcome assessment. Prospective data are extremely scarce. In this context, our multidisciplinary HHT group previously reported interim prospective findings suggesting that AT may be safely administered in selected patients without significant worsening of bleeding outcomes [18]. Here, we present updated results from this ongoing prospective cohort study conducted at a tertiary HHT referral center, providing one of the largest prospective cohorts with standardized longitudinal assessment of bleeding severity available on this topic.

## 2 Materials and Methods

Starting in January 2019, we conducted a prospective observational study at the HHT Center of Fondazione Policlinico Universitario A. Gemelli IRCCS, aimed at evaluating the safety of AT in patients with HHT. Inclusion criteria were (1) a definite diagnosis of HHT, (2) a new prescription of AT, and (3) provision of written informed consent. A definite diagnosis of HHT was established by either genetic confirmation or the presence of at least 3 of the 4 Curaçao criteria (recurrent spontaneous epistaxis; multiple mucocutaneous telangiectases; visceral AVMs; a first-degree relative with definite HHT). A new prescription of AT was defined as initiation of antiplatelet or anticoagulant therapy in patients who had not received any antithrombotic treatment in the previous 3 months. Upon enrollment, the baseline bleeding status of each patient was assessed in terms of Epistaxis Severity Score (ESS) [19], hemoglobin (Hb) levels, and transfusion requirements. These parameters were expressed as mean ± SD of the available values measured during the 3 months preceding initiation of AT.

Follow-up visits (in-person or remote) were scheduled at 1 and 3 months after enrollment, and every 3 months thereafter. The primary outcome was the incidence of hemorrhagic events during AT, classified according to the criteria of the International Society on Thrombosis and Haemostasis as major bleedings (MBs), clinically relevant non-MBs, and minor bleedings [20,21]. The main secondary outcome was worsening of epistaxis, assessed using the ESS. Worsening was defined as a shift to a higher severity category or by comparing mean ESS values during therapy with those recorded in the 3 months prior to treatment initiation. Additional secondary outcomes included a drop in hemoglobin levels of at least 1 g/dL after at least one month of therapy compared with baseline and increased transfusion requirements, evaluated after at least 3 months of therapy compared with the 3 months preceding AT initiation.

All patients were managed according to the standard multidisciplinary clinical practice of our HHT referral center, which includes regular otorhinolaryngology assessment and local treatment of nasal telangiectases whenever clinically indicated. Patients with anemia disproportionate to the severity of epistaxis or with a history suggestive of GI bleeding underwent endoscopic evaluation and treatment whenever appropriate. Individualized recommendations regarding bleeding prevention and a tailored follow-up plan were provided before initiation of AT.

The study was approved by the institutional Ethics Committee (Protocol No. 49901/18; ID: 2329), and all participants provided written informed consent. Statistical analyses were performed using SPSS 20.0 and GraphPad Prism 7.0. Continuous variables are expressed as mean ± SD, and categorical variables as counts and percentages. Paired Student’s *t*-test or Wilcoxon signed-rank test was used as appropriate. A *P* value < .05 was considered statistically significant.

## 3 Results

Since the beginning of the study, we enrolled 33 patients. The demographic and clinical characteristics of the study population are summarized in Table 1. The patients were mostly males (69.7%), with a mean age of 65.8 ± 12.9 years. All patients reported spontaneous episodes of epistaxis (100.0%). Thirty-two patients (97.0%) had mucocutaneous telangiectases and a positive family history of HHT. Pulmonary AVMs were present in 13 patients (39.4%), hepatic AVMs in 16 patients (48.5%), and 1 patient (3.0%) had a cerebral vascular malformation. A mixed phenotype (defined by the coexistence of visceral vascular malformations involving more than one organ) was present in 9 patients (27.3%). Twelve patients (36.4%) had a history of GI bleeding due to the presence of GI AVMs.

**Table 1:** Baseline characteristics of the population.

Mean age (years ± SD)	65.8 ± 12.9
White Caucasian (*n*/total)	33/33
Gender (males/females)	23/10
Epistaxis (*n*/total)	33/33
Mucocutaneous teleangiectases (*n*/total)	32/33
Family history of HHT (*n*/total)	32/33
Pulmonary AVMs (*n*/total)	13/33
Hepatic AVMs (*n*/total)	16/33
Cerebral AVMs (*n*/total)	1/33
Mixed phenotype (visceral vascular malformations in more than one organ) (*n*/total)	9/33
Previous gastrointestinal bleeding (*n*/total)	12/33

AVMs, arteriovenous malformations; HHT, hereditary hemorrhagic telangiectasia, *n*, number.

The 33 patients enrolled in the study received a total of 41 AT courses. As shown in Table 2, there were 16 courses of antiplatelet agents and 25 courses of anticoagulant agents. The most frequent indications for AT were venous thromboembolism (31.7%), atrial fibrillation (26.8%), and secondary prevention following cerebrovascular events (17.1%). The mean follow-up duration was 21.1 ± 19.2 months. Additional details regarding individual antithrombotic treatment courses, including the specific drug, prescribed dose, indication, and treatment duration, are reported in Supplementary Table S1.

**Table 2:** Type of AT, indication to AT, duration of the AT courses.

*Number of AT courses/number of patients*	41/33
Antiplatelet therapy (*n*/total of AT courses)	16/41
ASA (number of courses)	11
clopidogrel (number of courses)	4
indobufen (number of courses)	1
Anticoagulant therapy (*n*/total of AT courses)	25/41
DOAC (number of courses)	14
enoxaparin (number of courses)	7
fondaparinux (number of courses)	2
AVK (number of courses)	2
*Indication to AT courses*	
Venous thromboembolism (*n*/total of AT courses)	13/41
Atrial fibrillation (*n*/total of AT courses)	11/41
Secondary prevention after stroke/TIA (*n*/total of AT courses)	7/41
Secondary prevention after MI (*n*/total of AT courses)	6/41
Peripheral artery disease (*n*/total of AT courses)	3/41
Left atrial appendage occlusion (*n*/total of AT courses)	1/41
*Mean duration of follow-up (months ± SD)*	21.1 ± 19.2
Mean duration of antiplatelet therapy courses (months ± SD)	21.9 ± 16.1
Mean duration of anticoagulant therapy courses (months ± SD)	20.6 ± 21.3

AT, antithrombotic treatment; ASA, acetyl salicylic acid; AVK, antivitamin K; DOAC, direct oral anticoagulant; MI, myocardial infarction; *n*, number; TIA, transient ischemic attack.

As summarized in Table 3, during follow-up, there were 2 MBs (4.9%), both occurring during anticoagulant therapy. The first case involved GI bleeding during edoxaban therapy for atrial fibrillation, leading to severe anemia requiring multiple blood transfusions in a patient with a prior history of GI bleeding. The second case was severe worsening of epistaxis during apixaban therapy, leading to the need for blood transfusions and permanent discontinuation of treatment. Additionally, there were 3 clinically relevant non-MBs, 2 of them during antiplatelet therapy (both worsening of epistaxis requiring medical evaluation and temporary treatment suspension) and one during dabigatran therapy (hematuria in a patient with bladder pathology). Only one minor bleeding was recorded (hemorrhoidal bleeding in a patient on enoxaparin).

**Table 3:** Bleeding complications.

Major bleeding (*n*/total of AT courses)	2/41
– in subjects taking anticoagulants (*n*/total of anticoagulant courses) – in subjects taking antiplatelets (*n*/total of antiplatelet courses)	2/250/16
CRNM bleedings (*n*/total of AT courses)	3/41
– in subjects taking anticoagulants (*n*/total of anticoagulant courses) – in subjects taking antiplatelets (*n*/total of antiplatelet courses)	1/252/16
Minor bleedings different from epistaxis	1/41
(*n*/total of AT courses)	
– in subjects taking anticoagulants (*n*/total of anticoagulant courses) – in subjects taking antiplatelets (*n*/total of antiplatelet courses)	1/250/16
Mean Hb levels (gr/dL ± SD) during AT vs prior to AT	11.0 ± 2.5 vs 11.4 ± 2.5
Change in need for transfusion during AT (*n*/total of AT courses)	5/41
Worsening of epistaxis (*n*/total of AT courses)	10/41
– in subjects taking anticoagulants (*n*/total of anticoagulant courses) – in subjects taking antiplatelets (*n*/total of antiplatelet courses)	4/256/16
Mean ESS ± SD during AT vs prior to AT	3.4 ± 2.2 vs 3.2 ± 2.1

AT, antithrombotic treatment; CRNM, clinically relevant nonmajor; ESS, epistaxis severity score; gr/dL, grams/deciliter; Hb, hemoglobin; *n*, number.

Worsening of epistaxis, according to the ESS, was observed in 10 courses of therapy (24.4%), 4 on anticoagulants and 6 on antiplatelet agents. However, the mean ESS measured during AT was not statistically different from that measured at the time of enrollment (3.4 ± 2.2 vs 3.2 ± 2.1, respectively; 95% CI 3.2-1.5; *P* = .2988) (Table 3).

No significant changes were observed in mean hemoglobin levels during therapy compared with baseline (11.0 ± 2.5 vs 11.4 ± 2.5 g/dL, respectively; 95% CI -1.5–1.5; *P* = .9609) (Table 3).

A change in need of blood transfusions after starting treatment was observed in 5 courses of therapy (12.2%).

## 4 Discussion

For many years, HHT has been regarded as a purely hemorrhagic disorder. However, accumulating evidence indicates that thrombotic events occur with a measurable incidence in the HHT population and that bleeding manifestations coexist with a clinically relevant thrombotic risk in this disease [[8], [9], [10], [11]]. Although the mechanisms underlying the potential prothrombotic tendency in HHT remain uncertain, chronic iron-deficiency anemia, leading to elevated plasma levels of coagulation factor VIII, endothelial dysfunction, and systemic inflammation, is considered a major contributor [[8], [9], [10], [11]]. In this context, it is crucial to develop prophylactic and therapeutic strategies that balance hemorrhagic and thrombotic risks, thereby counteracting the reluctance to prescribe antithrombotic therapy to patients with HHT based solely on bleeding concerns.

The present study represents one of the largest prospective cohorts evaluating AT therapy in HHT and provides standardized longitudinal assessment of bleeding severity, Hb levels, and transfusion requirements. Our findings indicate that, in carefully selected patients managed within a specialized multidisciplinary setting, AT was not associated with substantial deterioration of overall bleeding burden.

Our findings should be interpreted in the context of the existing literature. Previous retrospective studies, including the VASCERN cohort and the RIETE registry, highlighted the challenges of AT in HHT by reporting worsening epistaxis, treatment discontinuation, and clinically relevant bleeding events [13,14]. More recent multicenter retrospective cohorts further emphasized the complexity of antithrombotic management in this population. In particular, Virk et al. reported that approximately half of treated patients required dose adjustment or discontinuation because of bleeding, with measurable effects on Hb levels and transfusion requirements [15]. Similarly, the RETROPLACOTEL study demonstrated that MB events clustered predominantly during the early phase of treatment and identified older age, previous GI bleeding, and recent transfusion requirements as predictors of poor treatment tolerance [16]. In our cohort, MB events also occurred exclusively during anticoagulant therapy and included a patient with a previous history of GI bleeding, in keeping with the risk factors identified in RETROPLACOTEL. At the same time, comparisons across studies should be interpreted cautiously. Differences in study design, patient selection, sample size, severity of HHT manifestations, burden of visceral vascular malformations, indications for AT, and organization of care may all substantially influence bleeding outcomes. In particular, our study was prospective and conducted within a specialized HHT center using standardized longitudinal assessment of bleeding severity, whereas most previous studies relied on retrospective data collection. Therefore, the observed differences in bleeding outcomes should not be interpreted as evidence of superior safety but rather as reflecting the distinct clinical settings and patient populations investigated. Nevertheless, our findings suggest that, in carefully selected patients managed within specialized multidisciplinary centers, worsening of epistaxis does not necessarily translate into clinically meaningful deterioration of overall bleeding burden, anemia, or transfusion requirements during short-term follow-up.

This study has limitations. First, the relatively small sample size reflects both the rarity of HHT and the limited number of patients in whom a new indication for AT arises over a relatively short period. The sample size also precluded meaningful subgroup analyses according to antithrombotic regimen or clinical phenotype. Second, HHT is characterized by considerable clinical heterogeneity, including differences in the distribution and severity of vascular malformations, bleeding phenotype, and overall disease burden. Consequently, our findings may not be generalizable to the full spectrum of patients with HHT requiring AT. Finally, although prospectively conducted, the study reflects the experience of a single tertiary referral center, where multidisciplinary expertise and close follow-up may have influenced clinical outcomes. Larger prospective multicenter studies are needed to confirm the generalizability of these findings.

## 5 Conclusion

The present study extends our previously published interim experience and currently represents one of the largest prospective cohorts available on AT in HHT, with longer follow-up and systematic longitudinal assessment of clinically relevant bleeding outcomes. Our updated prospective data suggest that AT can be safely administered in selected patients with HHT when managed in specialized centers with a multidisciplinary approach. Although careful monitoring is required—particularly in patients receiving anticoagulants or with a prior history of bleeding—the overall risk of MB appears acceptable and should not preclude the use of AT when clinically indicated. Our findings extend the limited prospective evidence supporting current guideline recommendations by demonstrating that standardized longitudinal assessment may provide a more comprehensive evaluation of treatment safety than retrospective reporting alone. Nonetheless, our findings should be considered supportive rather than definitive, and further large-scale prospective studies are warranted to better define optimal therapeutic strategies and identify patients at higher risk of treatment-related complications.
