# Supplementary material for: Safety of antithrombotic therapy in hereditary hemorrhagic telangiectasia: results from a prospective cohort study
Source: Res Pract Thromb Haemost. 2026 Sep 3;10(6):106943. doi: 10.1016/j.rpth.2026.106943 (PMC13634754; doi:10.1016/j.rpth.2026.106943)
Supplement: Supplementary file 1 [file mmc1.docx]

**Supplementary Table S1.** Individual AT courses included in the prospective cohort

| **Drug** | **Indication for treatment** | **Dosage prescribed** | **Treatment duration** |
| --- | --- | --- | --- |
| ASA | Secondary prevention after TIA | 100 mg o.d. | 34 months |
| ASA | Secondary prevention after TIA | 100 mg o.d. | 36 months |
| ASA | Secondary prevention after stroke | 100 mg o.d. | 39 months |
| ASA | Secondary prevention after stroke | 100 mg o.d. | 43 months |
| ASA | Secondary prevention after stroke | 100 mg o.d. | 39 months |
| ASA | Peripheral artery disease | 100 mg o.d. | 29 months |
| ASA | Peripheral artery disease | 100 mg o.d. | 22 months |
| ASA | Peripheral artery disease | 100 mg o.d. | 4 months |
| ASA | Secondary prevention after MI | 100 mg o.d. | 6 months |
| ASA | Secondary prevention after MI | 100 mg o.d. | 17 months |
| ASA | Secondary prevention after MI | 100 mg o.d. | 7 months |
| clopidogrel | Secondary prevention after MI | 75 mg o.d. | 48 months |
| clopidogrel | Secondary prevention after MI | 75 mg o.d. | 11 months |
| clopidogrel | Secondary prevention after MI | 75 mg o.d. | 5 months |
| clopidogrel | Left atrial appendage occlusion | 75 mg o.d. | 3 months |
| indobufen | Secondary prevention after TIA | 200 mg o.d. | 8 months |
| apixaban | Pulmonary embolism | 5 mg b.i.d. | 5 months |
| apixaban | Secondary prevention after pulmonary embolism | 2.5 mg b.i.d. | 31 months |
| dabigatran | Pulmonary embolism | 110 mg b.i.d. | 5 months |
| rivaroxaban | Secondary prevention after pulmonary embolism | 10 mg o.d. | 45 months |
| enoxaparin | Pulmonary embolism | 1 mg/kg b.i.d. | 1 months |
| enoxaparin | Pulmonary embolism | 1 mg/kg b.i.d. | 6 months |
| enoxaparin | Deep vein thrombosis | 1 mg/kg b.i.d. | 3 months |
| enoxaparin | Splanchnic vein thrombosis | 1 mg/kg b.i.d. | 3 months |
| enoxaparin | Splanchnic vein thrombosis | 1 mg/kg b.i.d. | 4 months |
| enoxaparin | Splanchnic vein thrombosis | 1 mg/kg b.i.d. | 4 months |
| enoxaparin | Splanchnic vein thrombosis | 1 mg/kg b.i.d. | 6 months |
| fondaparinux | Deep vein thrombosis | 7.5 mg o.d. | 3 months |
| fondaparinux | Deep vein thrombosis | 5 mg o.d. | 3 months |
| dabigatran | Atrial Fibrillation | 110 mg b.i.d. | 61 months |
| dabigatran | Atrial Fibrillation | 110 mg b.i.d. | 41 months |
| dabigatran | Atrial Fibrillation | 110 mg b.i.d. | 34 months |
| dabigatran | Atrial Fibrillation | 110 mg b.i.d. | 57 months |
| apixaban | Atrial Fibrillation | 5 mg b.i.d. | 39 months |
| apixaban | Atrial Fibrillation | 5 mg b.i.d. | 5 months |
| edoxaban | Atrial Fibrillation | 30 mg o.d. | 1 month |
| edoxaban | Atrial Fibrillation | 30 mg o.d. | 10 months |
| rivaroxaban | Atrial Fibrillation | 20 mg o.d. | 64 months |
| rivaroxaban | Atrial Fibrillation | 15 mg o.d. | 32 months |
| warfarin | Atrial Fibrillation | variable, based on INR | 13 months |
| acenocumarol | Secondary prevention after stroke in pAVM | variable, based on INR | 40 months |

AT: antithrombotic treatment; ASA: acetyl salicylic acid; b.i.d.: bis in die; INR: international normalized ratio; MI: myocardial infarction; o.d.: once daily; TIA: transient ischemic attack.
